# The Increasing Influence of Big Tech in Health and Medicine and the Need for a Public Health Ethics Perspective

**DOI:** 10.1093/phe/phaf005

**Published:** 2025-05-28

**Authors:** Steven R Kraaijeveld, Tamar Sharon

**Affiliations:** Department of Law, Ethics & Medical Humanities, Amsterdam University Medical Center, Amsterdam, the Netherlands; Department of Ethics and Political Philosophy and Interdisciplinary Hub for Digitalization and Society, Radboud University, Nijmegen, the Netherlands

## Abstract

Large consumer technology corporations are becoming increasingly influential in health and medicine. While this is sometimes beneficial to public health, it also raises many risks, like inequitable returns to the public sector in public-private medical partnerships or new dependencies on technology firms for the provision of public health goods and services. These risks are not always fully captured by existing frameworks. In this paper, we argue that it is time to adopt a public health ethics perspective on the increasing influence of Big Tech in health and medicine. A public health ethics perspective can not only capture the effects of Big Tech on the health of entire populations, but it also raises the important question of whether governments ought sometimes to intervene in the activities of Big Tech in order to safeguard public health.

‘[H]ealth [ … ] is a product of the human will, of a vigilance that must never falter’.

—Albert Camus, *The Plague*

## Introduction

The rapid growth of large consumer technology corporations (‘Big Tech’) over the past decades has been accompanied by Big Tech’s expansion into many different industries and fields, ranging from law and education to health and medicine (Stevens et al., 2022, 2024). In particular, the rise of digital and data-intensive health over the past years has paved the way for Big Tech to have an increasing presence in health and medicine (Sharon, 2016; Schuhmacher et al., 2023). To name but a few examples, large tech corporations (e.g. Alphabet, Apple, Amazon, Meta and Microsoft) have become involved in: home medical surveillance (Olsen, 2021); digital biomarkers (Verily, 2023); electronic health records (Microsoft, 2022), contact tracing, health tracking and predictive measures for infectious disease outbreaks (Barber, 2020; Siffels and Sharon, 2024); software and wearables for remote clinical studies (Apple, 2015); and, more generally, within the emerging fields of telemedicine, mHealth and eHealth (Iyengar, 2020; Bates, 2023).

These developments have raised various concerns. The activities of Big Tech in health and medicine have, for instance, been studied in relation to problems surrounding surveillance capitalism (Zuboff, 2019) and data colonialism (Ozalp et al., 2022; Sekalala and Chatikobo, 2023; Mejias and Couldry, 2024). In a recently completed project funded by the European Research Council between 2019 and 2024,^1^ we have taken another approach, namely by studying the effects of the so-called ‘Googlization’ of health from the perspective of ‘sphere transgressions’ (Walzer, 1983; Stevens et al., 2024). From this perspective, the increased digitalization of various societal spheres—such as health, education and law—invites new actors into spheres where they have not traditionally operated. These actors own the software and the hardware needed for digitalization and possess the relevant digital know-how and expertise, but they may not always share or heed, let alone promote, the core values of the spheres in which they come to operate. Understanding this phenomenon from the perspective of sphere transgressions highlights concerns and risks that are neither limited to traditional concerns about Big Tech (such as privacy and surveillance), nor to traditional concerns in health and medicine (such as patient safety and informed consent), but which have deeper effects on the spheres of health and medicine as such and on society more broadly. These risks include, for instance, undue influence on medical research agendas by private actors who are not domain experts; inequitable returns to the public sector in public-private medical partnerships; new dependencies on technology firms for the provision of public (health) goods and services; and a reshaping of the spheres of health and medicine in line with corporate values (Sharon, 2021; Sharon and Gellert, 2024).

As such, we believe that the growing presence of Big Tech in health and medicine constitutes what John Dewey (2016) has called a *public problem*, in that it raises the prospect of harms that are experienced collectively, if not individually. Given that the increasing influence of Big Tech in health and medicine frequently impacts, and poses risks to, not merely the health and well-being of individuals but also that of the public, we need a larger perspective from which to understand and ethically evaluate these developments. Indeed, the risks raised by Big Tech in health and medicine are often not fully captured by traditional regulatory mechanisms, which tend to be grounded in medical ethics and research ethics frameworks. Institutional review boards, for instance, often focus on harm to individual patients, for which principles like respect for autonomy, patient safety, confidentiality, and informed consent have importantly been developed (O’Neill, 2009). Similarly, ethical guidelines for clinical trials are historically based on ‘perceived therapeutic obligation to treat and benefit the patient‐participants’, without paying sufficient attention to duties to protect the population as a whole (Buchanan and Miller, 2006). Yet, many of the risks raised by the growing influence of Big Tech in health and medicine are of a collective and societal nature; they are not restricted to potential harms to individuals, be they patients or research participants.

In this paper, we argue that *public health ethics* provides an important perspective from which to evaluate many of the advancements surrounding Big Tech in health and medicine. Through a number of examples, we show that two distinctive characteristics of public health ethics^2^ are particularly valuable and well-suited to capture the risks associated with the expanding presence and power of large corporate actors and their products in health and medicine: 1) a focus on populations as the beneficiaries of health, and 2) a concern with the legitimacy of government interventions. Our claim is not that these two characteristics of public health ethics are the only relevant considerations when it comes to an ethical analysis of, for example, the digitalization of healthcare or the use of artificial intelligence (AI) in medicine. More modestly, we propose that public health ethics provide a currently underutilized yet crucial perspective from which to evaluate the ethics of these developments. It furthermore has the potential to inform and bridge both current and future discussions raised by the increasingly powerful and diverse roles that Big Tech actors are taking upon themselves in health and medicine.

## A Population Perspective

Due to the very nature of public health, public health ethics involves a distinctive public or population perspective (Holland, 2015; Dawson and Verweij, 2007; Turnock, 2015), which differentiates it from related disciplines like medical ethics (Jonsen, 1998) and distinguishes its focus from that of research ethics, where individual participants are the primary locus of ethical concern. Whereas in medicine, the patient is an individual person, in public health ‘the “patient” is the whole community or population’ (Beauchamp and Steinbock, 1999, 25). One of the central tenets of public health ethics is the recognition that the interests of individuals and groups are not always aligned. Tensions can and often do arise between the health preferences and choices of individuals and larger public health goals (Widdows, 2015; Faden et al., 2022).

A classic case where public health ethics dilemmas frequently occur is vaccination. Given that the benefits of an individual act of vaccination often extend beyond individual vaccinees, for example by preventing disease transmission or by contributing to group-level protection (Kraaijeveld, 2020), this can give rise to tensions between the health choices of individuals and larger public health aims (Dawson and Verweij, 2008). Individual health choices and behaviors—like vaccination decisions—usually have ramifications both for third parties (e.g. people with whom an individual comes into direct contact) and for society at large (e.g. on group-level immunity and disease transmission dynamics) (Kraaijeveld, 2023a; Kraaijeveld and Mulder, 2022). This is one reason why individual health choices can give rise to moral reasons to act (Kraaijeveld et al., 2024), and often take on acute moral significance, particularly during public health crises (Kraaijeveld and Jamrozik, 2022; Kraaijeveld, 2024).

Adopting a public health ethics perspective in the case of Big Tech entails asking what the effects of the activities of these actors are, or will be, at a population level—rather than merely at the level of individual patients or research participants. Even when there are potential benefits to individual patients or participants (e.g. when investments by Big Tech may contribute to diagnostic or therapeutic breakthroughs), wider effects on public health must be carefully considered.

There are a number of broader risks associated with the rise of Big Tech in health and medicine that are beyond the scope of medical ethics and that stand to negatively affect the public (Sharon, 2018). The increasing power of Big Tech can lead, for example, to corporations exerting unwanted influence on medical research agendas, which may advance corporate and personal rather than public interests. For example, Alphabet has heavily invested in Parkinson’s disease research in the past decade, with the development of hardware, software and over $1 billion funding for research. Sergey Brin, former head of Alphabet, has been open about the fact that the reason for this heightened interest and investment in Parkinson’s Disease is that a rare form of Parkinson’s runs in his family (Brin, 2008). While it can be argued that Brin’s interest in Parkinson’s is of value to public health, it is nonetheless a *personal* interest, and can therefore be seen as a form of philanthropy.^3^ Scholars have demonstrated that philanthropy, especially in fundamental areas of global and public health, can distort funding landscapes by funneling attention and resources to specific areas of interest (i.e. those about which philanthropists are concerned) while drawing goods away from other important areas of need (McGoey, 2015).

It cannot and must not be assumed that the (re)direction and (re)distribution of resources along the interests of powerful individual actors and corporations is straightforwardly good for public health. Even if the private and corporate distribution of health services may sometimes serve public health, personal and corporate interests may clearly also be unaligned with—and even directly opposed to—pressing public health needs. In this way, the influence of Big Tech on funding and research agendas can ultimately come at the expense of public health. A concrete example is the recent trend among Silicon Valley executives to explore means of life extension and anti-aging (Sample, 2022), which, as public and global health goals, are questionable at best—especially considering the scarce resources that are available to health care systems and the many burdens posed by (chronic) diseases.

Big Tech’s involvement in public health is nowhere more evident than in their recent efforts to address the spread of the COVID-19 virus. Companies like Facebook (now Meta), Microsoft, Alphabet, Amazon and Apple became involved in developing COVID-19-specific data collection, data sharing and data analysis tools; and, more generally, became active in funding research related to COVID-19. Verily (Alphabet’s life science subsidiary), for example, launched a screening and testing website and set up over 130 drive-through testing sites throughout the US.^4^ In the UK, Amazon, Microsoft, Google and Palantir were recruited early on in the pandemic to assist the National Health Service (NHS) in tracking data about hospital beds, oxygen capacity and ventilators (Fitzgerald and Crider, 2020). As discussed earlier, one of the most common patent problems raised by Big Tech operating in public health is a conflict of interests, which should always be considered as a potential ethical problem. In addition, the intermingling of Big Tech in public health can also lead to a clash in expertise, given that these technology corporations are not domain experts. A telling example is Google and Apple’s involvement in automated COVID-19 contact tracing. At the outbreak of the pandemic, in early 2020, numerous countries sought to develop smartphone applications that would be able to notify users who had been in close proximity with positive-testing individuals, thus relieving some of the burdens of manual contact tracing faced by already struggling healthcare systems (Ferretti et al., 2020). An ongoing discussion at the time centered on the privacy risks raised by automated contact tracing (Parker and Kind, 2020). On the one hand, privacy advocates called for apps to be developed with ‘decentralized’ data collection—meaning apps that locally store data on users’ phones rather than on a centralized data repository, where third parties might easily access it. On the other hand, some public health experts, epidemiologists and virologists called for the development of apps with ‘centralized’ data collection, given that centralized data storage would allow for better oversight of the collected information, including the identification of transmission trends and clusters of infection (Kelion, 2020).

In April 2020, in the midst of these discussions, Google and Apple jointly launched an application programming interface (API) for Apple and Android phones on which different contact tracing apps could run. The API would only work with decentralized apps, thereby putting an abrupt end to the discussion about the relative benefits and disadvantages of centralized versus decentralized storage. Google and Apple made decentralization a non-negotiable criterion for countries to run apps on their software, thus deciding the technicalities of this large-scale public health intervention (with accompanying regulatory and ethical concerns) *over and above* some public health experts and sovereign states (Sharon, 2020). Indeed, countries that had already developed national centralized apps (e.g. Germany, Norway and the UK) had to redesign those apps in order for them to work with Google and Apple. While Big Tech’s involvement happened to have been good for the privacy-friendliness of the intervention in this case, the benefits for public health—that is better oversight of infection transmissions through centralized storage—were questionable. The outcome was, in any case, not the result of informed (public) deliberation, but of Big Tech actors Google and Apple dictating the terms of use.

Another relevant example is the increasing digitalization of health care, as in in the case of eHealth and mHealth technologies, with AI tools that are often run by major technology corporations (De Raeve et al., 2016; Sax et al., 2018). Digital tools like AI-powered virtual assistants are increasingly being considered and implemented in health care practices, not only for the general population but also for specific patient groups (e.g. people with type 2 diabetes) (Buinhas et al., 2019; Gurtis et al., 2021). Big Tech is taking a keen interest in these developments, with Google having recently developed a medical Large Language Model (LLM) that can converse intelligibly (and apparently empathetically) with users (Tu et al., 2024). Aside from the above-mentioned issues associated with agenda setting, the use of generative AI tools within health care and medicine has wider implications. There are, for instance, specific populations that are at risk of being systematically ‘left behind’ by these developments, like those who are less able to use such tools (e.g. people with relatively low digital and literacy skills). These populations are less likely to be able to use these new technologies compared to other groups, which raises larger concerns about justice and access to healthcare resources (Kraaijeveld et al., 2025). The digitalization of care is accelerated not only by rising health care costs, but also by the profit- and market-concerns of Big Tech, which again raises questions about what it means for patient populations and for society more generally when Big Tech-driven technological innovations give rise to health inequalities (Weiss and Eikemo, 2017). From a global health perspective, disparate investments in, and unequal access to, medical and health care technologies created and operated by Big Tech may also create and exacerbate global health inequalities, which Big Tech itself is unlikely to have strong incentives to address.

As these examples indicate, the increasing power of Big Tech in health and medicine can lead to corporations exerting undue influence on medical research agendas, public health interventions and health care, which may come at the expense of public health. It should be clear by now that the corporate and personal interests of Big Tech actors are not straightforwardly aligned with public interests, and that their stewardship—even for seemingly beneficial public health projects—can be capricious. These developments can lead to a reshaping of the health sector according to corporate values and expertise (Sharon, 2018), which may be incongruent with the aims and values of public health, like a fair distribution of scarce healthcare resources.

It is crucial to see these risks as falling within the purview of public health ethics. After all, public health is an important societal good (Von Heimburg et al., 2022). Relying on ethical frameworks that focus on individual patients or research participants can obscure larger ethical concerns that arise at the level of populations. A population perspective allows us to see more clearly that overarching and cumulative effects on public health by Big Tech actors need to be carefully considered and may ultimately pose unacceptable risks to public health. The population perspective that a public health ethics approach provides is therefore much needed to inform discussions about risks surrounding the increasingly pervasive and powerful presence of Big Tech in health and medicine.

As for the question of what ought to be done when Big Tech poses unacceptable risks to public health, we now turn to a second important characteristic of public health ethics.

## The Legitimacy of Government Interventions

Since public health is inherently directed toward promoting the health of populations rather than individuals, not only do ethical questions about public health justice take center stage (Powers and Faden, 2006), but the question of who is responsible for safeguarding public health also inevitably arises. In light of this, another major concern of public health ethics is the potential for, and the moral legitimacy of, state interventions in the interest of public health (Faden et al., 2022). Governments have a moral responsibility to protect at least some collective health projects (Verweij and Houweling, 2014). Under one prominent conception of public health, public officials must take ‘appropriate measures pursuant to specific legal authority, after balancing private rights and public interests, to protect the health of the public’ (Rothstein, 2002, 146).

In the previously discussed case of vaccination, the question arises whether and to what extent governments are ethically justified in interfering with individual vaccination decisions (Kraaijeveld, 2023b), thus influencing and potentially restricting what would otherwise be autonomous health choices. One central principle that can potentially justify government interventions is the harm principle—originally formulated by the English philosopher John Stuart Mill—which holds that preventing harm to others is a necessary, if not sufficient, condition for restrictions of individual freedom (Mill, 1859/2005).

Relating these ideas back to the present discussion about the influence of Big Tech in health and medicine, a question that should be foregrounded is the following: To what extent ought governments to intervene in the activities of Big Tech corporations for the sake of public health?

As in many other public health ethics discussions, the harm principle can offer a rough answer. When the activities of Big Tech in health and medicine pose unacceptable risks of harm to public health, then this provides a *prima facie* moral reason for governments to intervene (e.g. through regulation and legislation). At the same time, the potential benefits to individual patients, participants and even larger groups at the hands of Big Tech should, of course, still be considered. These remain morally relevant. Our claim is precisely that a larger perspective that *also* includes collective or population health risks is necessary for a more comprehensive ethical analysis of the influence of Big Tech in health and medicine, which a public health ethics perspective is in a unique position to offer.

Questions about the legitimacy of government interventions, which are part of public health ethics, should therefore be extended to Big Tech actors in health and medicine, particularly while recognizing that, in principle, governments—rather than corporations—have a responsibility to protect the health of citizens (Gostin, 1986; Verweij and Houweling, 2014). Where interests clash—as in parallel debates about conflicts between individual health choices and wider public health interests—careful ethical analysis becomes all the more indispensable. As the capacities of Big Tech corporations to influence public health grow on a global scale, ethical questions about the role of governments in relation to these corporate actors are only bound to become more acute. Simple acquiescence by governments and public health officials does not seem to be desirable from an ethical perspective.

Another tangible example of where government intervention may be warranted is in the case of ‘paying twice’, which occurs when governments pay corporations for health data or services for which they have already paid (e.g. through public funding) (Wolitz, 2019). One reason for technology corporations to move into the sphere of health and medicine is to gain access to medical datasets that they do not own themselves, but that are needed to train algorithms and other AI-based medical decision support tools. These algorithms and services, once developed, are proprietary—even though they have been trained on publicly funded datasets—and can be sold back to the public sector. This raises ethical concerns about fair value sharing in partnerships with technology companies; it is also an explicit public issue in the sense that, whatever the benefits to individual patients, the public funds that are ‘lost’ by paying twice for the same product might have been used to promote public health in various other ways, which is especially regrettable given the scarcity of public funds. While the products generated by these collaborations would perhaps not have otherwise materialized, that does not automatically mean that paying twice is ethically justified or desirable from a public health perspective. In other words, wider public health consequences ought to be assigned due weight within a more comprehensive ethical analysis of public-private collaborations in health and medicine; and governments may sometimes have to intervene to prevent Big Tech practices that conflict with the interests of public health.

There is a serious risk that governments presently do not see (or do not wish to see) the extent to which they stand to receive an unfair share of value in partnerships with Big Tech. For example, as mentioned previously, during the COVID-19 pandemic the NHS allowed Google, Amazon, Microsoft and Palantir to access NHS data to train AI models. This was in exchange for data analytics services; but, at least originally, these corporations were granted property rights to these models (Fitzgerald and Cider, 2020), which would mean that the NHS would subsequently need to pay for access to the data.

Similarly, and on an even larger scale, the European Health Data Space (EHDS), which was approved by the European Parliament in April 2024, seeks to make the health data of European citizens accessible to health professionals across the EU and to make these data available to third parties for the purpose of research and innovation. Indeed, access by technology companies to the EHDS in order to develop AI-based medical decision support tools is explicitly stated as an example of its second aim.^5^ There are currently no specifications in the regulation, however, as to how the value from the development of such applications might flow back to European healthcare organizations or European citizens (Marelli et al., 2023). Here, too, the labor and costs that were involved in building the EHDS are carried by the public sector, while the value that Big Tech ultimately derives from access to it is privatized. In this case, the matter of government intervention arises because governments ought to ensure that value is justly distributed across the public and private sectors. This could be achieved, for instance, by charging access to datasets, by specifying reduced costs of services, or by setting other conditions for collaboration (Mazzucato, 2018).

Finally, a focus on whether and when government intervention is morally legitimate can also play a valuable role in analyzing and addressing an additional risk of Big Tech expansionism into health and medicine, which is the emergence of new dependencies on Big Tech by public healthcare systems for the provision of health services and medical research, both of which are basic public goods (Anomaly, 2011). It is misleading to see the health and medical services of these corporations as standalone products; they are almost always part of a broader ‘suite’ of hardware, software, apps, clouds and operating systems. Using any one of these services generally implies buying into an entire ecosystem in which products from other providers cannot function, thus creating technological lock-in (Balayn and Gürses, 2021). Most importantly, we should see these services as beginning to form the computational infrastructure that digital health and medicine will be running on in the future. A growing dependency on Big Tech actors for the provision of these services makes public healthcare systems particularly vulnerable to the whims and interests of actors who do not necessarily share the values and norms that underpin healthcare, or the more general moral principles of care in most Western democracies: access for all, based on need. As basic public goods, health provision and medical research are goods that, when threatened, may justify interventions by governments. The second characteristic of public health ethics, namely a concern with the legitimacy of government restrictions in the interest of collective health goals, allows us to see this. It raises the possibility that governments ought sometimes to intervene in the activities of Big Tech in order to safeguard public health.

## Conclusion

We have argued, using a number of examples, that public health ethics provides an important and much-needed perspective to understand and ethically evaluate the novel risks posed by Big Tech’s increasing influence in health and medicine. We contended that two distinctive characteristics of public health ethics—a focus on populations as the beneficiaries of health, and a concern with the prospect and legitimacy of government interventions for the sake of public health—allow us to better see and confront those risks. By reframing the pervasive presence of Big Tech in health and medicine, and in data-intensive health and medicine more generally, as a public health ethics problem, we hope to contribute to ongoing discussions about what constitutes and serves good public health in the age of Big Tech.

## Footnotes

1 Grant number: 804985.

2 It should be noted that there is much discussion about the distinctiveness of public health ethics as a field; to what extent its concerns overlap with related fields like medical ethics; and so on. We do not wish to take a strong stand on this matter here. There is bound to be some overlap between public health ethics and related disciplines. However, we concur with and follow Ruth Faden, Justin Berstein, and Sirine Shebaya (2022) in seeing a population focus and the role of government in potential public health interventions as two key and distinctive characteristics of public health ethics.

3 Brin channels most of his Parkinson’s research funding through his philanthropy, ‘Aligning Science Across Parkinson’s’.

4 For an overview, see: https://www.projectbaseline.com/studies/covid-19/

5 See, e.g. question no. 7, example 2, on the ‘Questions and answers’ page: https://ec.europa.eu/commission/presscorner/detail/en/QANDA_22_2712
